# Use baseline axial length measurements in myopic patients to predict the control of myopia with and without atropine 0.01%

**DOI:** 10.1371/journal.pone.0254061

**Published:** 2021-07-15

**Authors:** Loreto V. T. Rose, Angela M. Schulz, Stuart L. Graham

**Affiliations:** Faculty of Medicine and Health Sciences, Macquarie University, Macquarie Park NSW, Australia; Cairo University Kasr Alainy Faculty of Medicine, EGYPT

## Abstract

**Purpose:**

Identifying axial length growth rate as an indicator of fast progression before initiating atropine 0.01% for myopia progression in children.

**Method:**

From baseline, axial length growth over six months was measured prospectively. Subjects were then initiated on atropine 0.01% if axial length growth was greater than 0.1mm per 6 months (fast progressors), axial length and spherical equivalent change measurements recorded every six months. The rate of change was compared to the baseline pre-treatment rate. If axial length change was below the threshold, subjects received monitoring only.

**Results:**

73 subjects were identified as fast progressors and commenced atropine 0.01%, (mean baseline refraction of OD -2.9±1.6, OS -2.9±1.8 and a mean baseline axial length OD 24.62 ± 1.00 mm, OS 24.53 ± 0.99 mm). At six months, the mean paired difference of axial length growth rate was significantly reduced by 50% of baseline (all 73 subjects, p<0.05). 53 subjects followed to 12 months, and 12 to 24 months maintained a reduced growth rate. Change in mean spherical equivalent was significantly reduced compared to pre-treatment refractive error (mean paired difference p<0.05) and at each subsequent visit. 91 children were slow progressors and remained untreated. Their axial length growth rate did not change significantly out to 24 months. Spherical equivalent changed less than -0.5D annually in this group.

**Conclusion:**

Identifying fast progressors before treatment initiation demonstrated a strong treatment effect with atropine 0.01% reducing their individual rate of myopia progression by 50%. Another large group of myopic children, slow progressors, continued without medical intervention. A baseline axial length growth rate is proposed as a guideline to identify fast progressors who are more likely to benefit from atropine 0.01%.

## Introduction

The prevalence of myopia is increasing worldwide, with pathological myopia (defined by the WHO as -5.0 D or higher) a growing cause of vision loss [1, 2]. Pathological myopia results in an increased risk of glaucoma, retinal detachment, choroidal neovascularisation and macular disease [3, 4]. Studies targeting myopia progression intervention have found that atropine eye drops consistently reduce progression as a dose dependant response [5–10]. The recent LAMP study has noted that atropine 0.05% had the most significant effect in retarding both myopic dioptric progression and axial elongation [8, 9]. However, this dose did result in greater pupil dilation and loss of accommodation. A previous study has noted that doses greater than atropine 0.02% will result in significant clinical symptoms of accommodation paralysis and pupil dilation [11].

Low dose atropine 0.01% is at the forefront [6, 7, 10, 12–14], with recent reviews [1, 15] advocating its use for myopia progression due to its high tolerance, minimal side effects and lowest rebound effect after treatment [12]. To date, there is limited published data on the use of atropine 0.01% in the clinical setting and no consensus as to when myopic children should commence treatment [16]. There is also controversy about whether atropine 0.01% has enough of an effect on axial length AL) growth which is the target of myopia progression [8–10, 12, 17].

Historical data suggested that an average childhood AL growth be 0.1mm per 6 months [6, 18]. An analysis of average axial change in Europe is variable with age with greater progression in under 9 years [19]. There is also an ethnic difference with greater progression noted in the Asian population (average of 0.30 mm/year) [9, 20, 21]. Various studies have used dioptric progression (usually > 0.5D/year) to define fast progressors to initiate myopia intervention treatment [22, 23]. Given that an axial elongation of 0.20 mm equates to approximately 0.5D [24], the cut off to define fast progressors was a growth rate exceeding 0.10 mm/ 6 months. The current study is in the clinical setting and utilizes serial baseline AL measurements to determine an AL progression rate before treatment. Treatment with atropine 0.01% were initiated on fast progressors with six-monthly follow up. AL and refractive change rates were compared before and after initiating treatment and followed over 6 monthly intervals.

## Materials and methods

This is a prospective cohort study of myopic children presenting to clinical practice for ophthalmic review. Participants were referred to clinical practice for an eye examination, usually concerned with myopia and myopia progression. Written informed consent was obtained from parents or guardians. The study was approved by the Human Research Ethics Committee at Macquarie University and conducted according to the tenets of the Declaration of Helsinki. Participants were required to be old enough to cooperate with accurate AL measures to enter the study with any myopia and astigmatism level. Patients with ocular disease such as retinal disease, previous use of other methods for myopia control, or known allergy to atropine or known systemic disease were excluded from the study.

**Fig 1:**
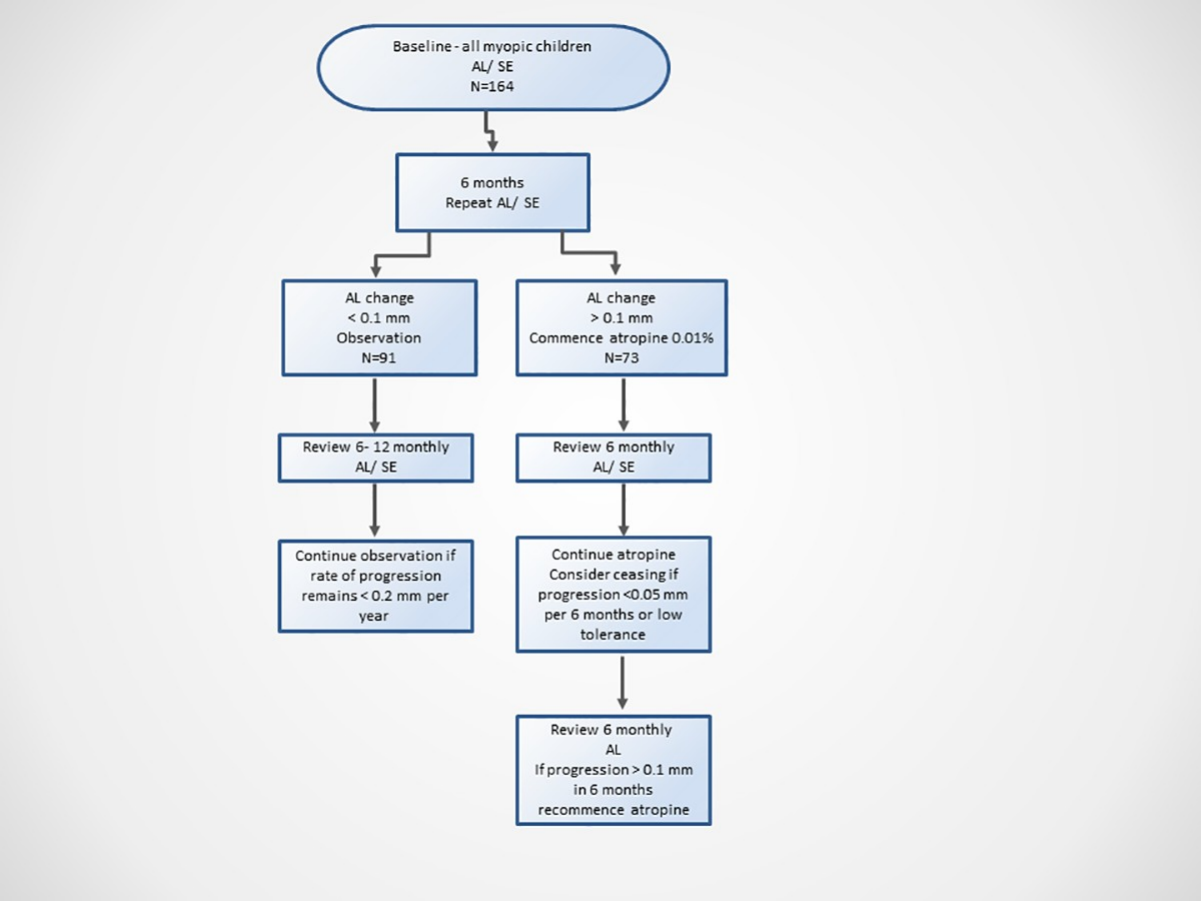
Participation flowchart in atropine 0.01% study. Legend: AL = axial length, SE = spherical equivalent.

Baseline review included best-corrected vision, cycloplegic retinoscopy refraction resulting in 6/6 vision (Snellen), duochrome red/green testing with this prescription to ensure accurate refraction. Fundus examination and AL (IOLMaster, Carl Zeiss Meditec) was performed. A high signal to noise ratio (individual signal to noise ratio greater than 2) is an essential measure of accuracy in each measurement [25]. The signal to noise ratio was high, often 200 to 500, with the only variable being fixation as no lens opacity was present in the participants. The AL measurement was observed for an ideal graphical display with maximum narrow peaks and multiple measurements with at least 4 within 0.02 mm. Lifestyle information was given regarding increased sunlight exposure and the reduction of recreational near activities. All participants received the same counselling at the first visit before establishing the baseline, and similar counselling was given at each subsequent visit. Glasses were updated to the full myopic script. Fig 1 demonstrated a participant flowchart. Participants were reviewed at six-month intervals with repeat vision, refraction (either cycloplegic or subjective dependent on age to achieve 6/6 vision Snellen) and AL measure. If AL was observed to increase by more than 0.1mm per 6 months (fast progressors), atropine 0.01% one drop nightly in each eye was initiated with an ongoing six-monthly review. At follow-up, participants and parents were questioned regarding any side effects of atropine therapy, especially any glare or near blur, medical illness and compliance issues. Change in AL and refractive error was analyzed at each review and compared to the baseline measurements. Treatment was initiated with a plan to continue for a minimum of 2 years and only ceased if AL growth was consistently very low after this period (<0.05 mm/6 months). If the myopic children did not reach the AL change treatment threshold (slow progressors), they continued to be monitored at 6–12 month intervals with AL change measurements and refractive change (spherical equivalent: SE) recorded. If a slow progressor was reviewed, and in the preceding six months, they had reached the threshold (0.1mm per 6 months), then atropine 0.01% was initiated to best manage myopia progression.

### Statistical analysis

The mean values of ocular parameters were calculated for both eyes. Changes to parameters were calculated by the difference between the baseline visit and the designated follow up visit of the treatment group. Data from participants who had poor compliance were excluded from the analysis. Poor compliance was defined as using other myopia control interventions or stopping the drops for more than two weeks.

Two independent sample t-tests were used to compare parameters at baseline and follow-up period with p values for the mean paired difference calculated (Minitab Express Version 1.5.2). Chi-squared analysis for the frequency distribution of baseline ethnicity was conducted (GraphPad Prism Version 7.02).

## Results

A total of 164 myopic children 4–17 years old were monitored. A qualifying fast AL growth rate was observed in 73 patients, mean age nine years old (4–15 years old), a mean baseline refraction of OD -2.9±1.6, OS -2.9±1.8 with a mean baseline AL growth rate of OD 24.62 ± 1.00 mm, OS 24.53 ± 0.99 mm. The demographics of the total participants were compared (Table 1). The fast progressor group mean age was just over one year younger (statistically significant p<0.05) with a higher proportion of females. The initial AL and SE in the fast progressor group were similar but slightly higher in both parameters. The fast progressor group had a greater proportion of East Asian ethnicities than the broader ethnic distribution in the slow progression group (Table 1).

**Table 1:** Baseline demographics.

Presentation Demographics	Slow progressor (n = 91) (SD)	Fast progressor before treatment (n = 73) (SD)
mean Age (year)	10.59 (3.16)	9.21 (2.22) *
Male gender no.	49	34
Ethnicity no.		
East Asian	12	35
Indian	29	13
Middle East	23	12
Caucasian	19	9
Other/Combination	8	4
Mean AL (mm)		
OD	24.20 (1.29)	24.62 (1.01) ^†^
OS	24.18 (1.32)	24.53 (0.98) ^†^
Mean SE (D)		
OD	-2.57 (2.69)	-2.95 (1.61) ^†^
OS	-2.66 (2.79)	-2.85 (1.76) ^†^

AL = axial length; SE = spherical equivalent; D = diopter; SD = standard deviation; OD = right eye; OS = left eye.

* t-test statistically significant difference p<0.05

^†^ t-test not statistically significant difference p>0.05.

At six months follow up, the fast progressor group had a mean paired difference of AL growth rate significantly reduced to around half recorded before the treatment initiation (mean AL change OD 0.13±0.13 mm, OS 0.11±0.14 mm, p< 0.05). The reduced growth rate in the 53 subjects followed over 12 months and maintained in the 27 subjects followed to 24 months (Table 2). Mean SE change at every six-monthly interval was similarly reduced, and the mean paired difference compared to before treatment was statistically significant (p<0.05). Similar statistically significant results were found at each subsequent six-monthly follow-up (Table 2). Smaller numbers followed up to 48 months had a similar trend for both AL and SE. Treatment was continued for a minimum of two years and continued beyond this if axial growth exceeded 0.05 mm per 6 months. Currently, three subjects have ceased atropine therapy after 24 to 30 months on treatment without a rebound in AL growth to 12 months follow up. A further two subjects have restarted atropine 0.01%, with an increased growth rate greater than 0.1 mm after a six-month break in atropine treatment.

**Table 2:** Progression of atropine 0.01% treatment group.

Review period and subject numbers	Eye	Mean ΔAL (mm)(SD)	MPD (SD)	Mean ΔSE(D)(SD)	MPD (SD)
6 months before treatment n = 73	OD	0.27 (0.13)		-0.56 (0.52)	
	OS	0.24 (0.16)		-0.53 (0.49)	
6 months atropine 0.01% n = 73	OD	0.14 (0.10)	0.13 (0.13) ***	-0.27 (0.66)	-0.29 (0.94) *
	OS	0.13 (0.13)	0.11 (0.14) ***	-0.20(0.33)	-0.33 (0.66) ***
6–12 months treatment n = 53	OD	0.13 (0.10)	0.15 (0.15) ***	-0.26 (0.39)	-0.37 (0.67) **
	OS	0.12 (0.10)	0.11 (0.17) ***	-0.21 (0.36)	-0.33 (0.58) ***
12–18 months treatment n = 38	OD	0.10 (0.10)	0.18 (0.15) ***	-0.18 (0.40)	-0.47 (0.68) **
	OS	0.09 (0.10)	0.12 (0.19) **	-0.16 (0.34)	-0.38 (0.57) **
18–24 months treatment n = 27	OD	0.12 (0.08)	0.14 (0.12) ***	-0.21 (0.37)	-0.40 (0.49) **
	OS	0.10 (0.07)	0.14 (0.11) ***	-0.08 (0.40)	-0.49 (0.61) **
24–30 months treatment n = 16	OD	0.08 (0.08)	0.18 (0.12) **	-0.17 (0.32)	-0.43 (0.57) *
	OS	0.09 (0.07)	0.15 (0.10) ***	-0.27 (0.32)	-0.34 (0.55) *
30–36 months treatment n = 9	OD	0.11 (0.04)	0.13**	-0.17 (0.25)	-0.50^†^
	OS	0.09 (0.03)	0.14**	-0.17 (0.22)	-0.50^†^
36–42 months treatment n = 6	OD	0.14 (0.07)		-0.50 (0.76)	
	OS	0.13 (0.07)		-0.21 (0.33)	

AL = axial length; SE = spherical equivalent; D = diopter; SD = standard deviation; OD = right eye; OS = left eye. MPD = mean paired difference

*** t-test statistically significant difference p<0.0001

** t-test statistically significant difference p < 0.005

* t-test statistically significant difference p < 0.05

^†^ 95% confidence t test Mann Whitney.

The atropine drops were well tolerated, with no subjects reporting side effects due to the drops, such as difficulty with glare/photophobia or near blur was communicated between visits or at their follow up clinic appointment. There were ten participants excluded in the treatment group analysis who were either lost to follow-up (six participants), initiated other treatment such as orthokeratology (one participant), or poor compliance with no treatment for greater than two weeks between the first review period (three participants).

**Fig 2:**
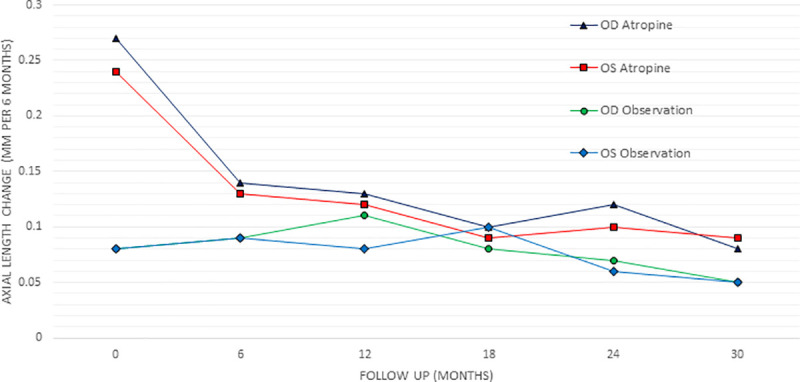
Graph showing change in the axial length in the observation and atropine 0.01% group over each six months.

**Fig 3:**
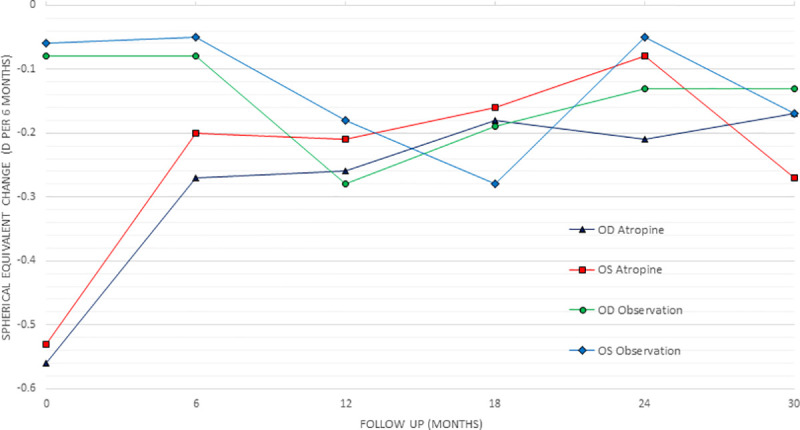
Graph showing change in the spherical equivalent in the observation and atropine 0.01% group over each six months.

A total of 91 slow progressors were monitored, mean age eleven years old (4–17 years), mean baseline refraction of OD -2.6±2.7, OS -2.7±2.8. The mean AL growth at six months was OD 0.08±0.07mm, OS 0.08±0.08mm. The mean total AL growth over the first and second 12 months of the observation group did not change significantly. Similarly, mean SE changed less than -0.5D in each year of testing in the slow progression group (Table 3). Comparing the slow progression group to fast progressors on atropine 0.01%, the treatment group still progressed on average more at every interval in both AL and SE (Table 3), demonstrating the myopia progression is slowed but not totally controlled. Figs 2 and 3 show the progression rates for all groups graphically.

**Table 3:** Progression rate of observation and treatment group.

Growth rate	Slow progressors (observation) Mean (SD)	Fast progressors (atropine) Mean (SD)
AL (mm)		
Baseline-6 month	n = 74	n = 73
OD	0.08 (0.07)	0.14 (0.10)
OS	0.08 (0.08)	0.13 (0.13)
Annual change: Baseline-12 month	n = 62	n = 53
OD	0.15 (0.09)	0.28 (0.18)
OS	0.15 (0.09)	0.25 (0.21)
Annual change: 12–24 month	n = 18	n = 27
OD	0.13 (0.12)	0.22 (0.17)
OS	0.15 (0.08)	0.17 (0.12)
SE (D)		
Baseline-6 month	n = 74	n = 73
OD	-0.08 (0.27)	-0.27 (0.66)
OS	-0.06 (0.69)	-0.20 (0.33)
Annual change: Baseline-12 month	n = 62	n = 53
OD	-0.14 (0.33)	-0.50 (0.80)
OS	-0.15 (0.30)	-0.34 (0.49)
Annual change: 12–24 month	n = 18	n = 27
OD	-0.19 (0.38)	-0.42 (0.42)
OS	-0.28 (0.44)	-0.30 (0.49)

AL = axial length; SE = spherical equivalent; D = diopter; SD = standard deviation; OD = right eye; OS = left eye.

## Discussion

This prospective cohort study of myopic children presenting to clinical practice demonstrates that atropine 0.01% use was well tolerated and can be prescribed and appropriately managed in the clinical setting. This study treatment guideline helped define fast progressors based on AL elongation rate, who were considered most likely to benefit from treatment. There are no clear clinical guidelines for when to initiate or how long to treat with atropine for myopia progression [16]. This study is the first use of axial growth before any myopia treatment as a guide to begin and continue atropine 0.01% treatment in myopia progression in children. A recent publication treated poor responders to orthokeratology for a year (progressing greater than 0.3mm/year) with additional atropine 0.01% drops and demonstrated no effect [17]. AL measurement is a fast and objective measure of progression. It provides an additional marker of treatment response to determine in the clinical setting if the atropine dose is adequate, complimenting refractive outcomes. AL can help determine when to initiate and when to stop or recommence treatment.

In this study, the cohort treated was mean age nine years 4–15 years), all of whom were progressing faster than 0.10 mm to enter the treatment arm. Atropine 0.01% reduced AL growth by 50% compared with the baseline growth rate over a 6-month interval. The reduction in progression was seen over an extended follow-up and mirrored in elongation and dioptre change. This result is comparable to the ATOM studies five-year data [26]. The ATOM studies found that atropine reduced dioptric myopic progression in a dose response trend. When a washout year of no treatment was included, the lowest dose tested (0.01%), resulting in the least myopic progression overall in 3 years of the study [26]. A recent retrospective European study treated myopic children progressing more than 0.5 D/year with atropine 0.01% [22]. The treatment group demonstrated a 50% reduction in power progression over a year compared to progression before treatment and to a control group which was statistically significant. Another study, multicenter randomized, double-masked placebo control, confirmed the efficacy and safety of atropine 0.01% in a two-year study [27]. This Japanese study also demonstrated a statistically significant reduction of AL and SE myopic progression with atropine 0.01%. The current study demonstrated a high reduction in axial progression, and this may be due to only treating the faster progressors, resulting in a greater effect than on all myopic children in the randomised control trial.

Recent reports have demonstrated that intermediate doses of atropine may be more effective at retarding AL growth and hence myopia progression [8, 9]. The LAMP study found that atropine 0.05% reduce AL growth by half than placebo [8, 9]. This study also found loss of accommodation amplitude and dilation more common in the higher dose [8, 9]. A previous report noted that a dose greater than atropine 0.02% result in clinical symptoms due to accommodation paralysis and dilation [11].

It may be possible to adopt a step like treatment approach if further reduction in AL growth rate is warranted using a higher dose such as atropine 0.02% or 0.05%. If treatment response is monitored with AL measurements compared to baseline growth, individual treatment choices can maximize myopic growth control. This individual treatment is specifically essential in children with light coloured eyes who are more likely to experience glare and near blur at the higher doses of atropine.

There is some controversy regarding the effect of atropine 0.01% on AL growth. The atropine effect in myopia progression reduction in children is from slowing the AL growth and not related to corneal or lenticular changes [28]. The ATOM studies quote AL growth in the atropine 0.01% group to be 0.41 mm in two years [12]. In contrast, the LAMP study found 0.59 mm AL growth in 2 years in the atropine 0.01% group [8]. In the current study, the mean AL progression in the treatment group was right eye 0.45 mm and left eye 0.37 mm of the participants who reached two years (similar to the ATOM study). Considering this group were the fast progressors, and on average, demonstrated double this growth rate before atropine 0.01% treatment and maintained reduction in follow up, this is a clinically significant result. Our treatment group contained a more significant East Asian population in keeping with epidemiological data, consistent with the observation that this population may have a higher prevalence of myopia than the Caucasian population due to genetic and cultural differences [29–31].

Epidemiology studies have found that the average AL growth rate is as high as 0.70 mm in two years in Shanghai [32]. Other studies, such as the ATOM studies, found a 0.4 mm change in 2 years in the placebo group [6]. There is also evidence that AL growth continues beyond 13 years, as previously suggested [33]. More recent data suggest that eye growth continues between 13 and 18 years of age [34]. The current study demonstrates that in myopic children, eye growth can continue into the teenage years significantly and that growth can be moderated with atropine 0.01%. The study also identified a large group of myopic children, including some as young as four years old, who show slow progression and can be clinically monitored without medical intervention. The LAMP study recently published the effect of different atropine doses on age and found the younger cohort were poorer responders and required higher concentration to achieve a similar reduction in myopic progression than in the older children in the study [35]. This report adds weight to monitoring ocular axial growth at a young age to assist when to treat and review the treatment effect.

In the current study, 11% of the participants on atropine 0.01% who reached two years, progressed by more than -1.5 D, and 7% progressed by -2.0 D or more. The rate of non-responders in the ATOM studies (defined as progression of 1.5 D or higher over the first two years) was almost 10% [26]. In the LAMP study, 19.2% of participants on atropine 0.01% progressed by 2 D or more in two years [8]. The definition of non-response of dioptre progression of more than -1.5 D per year may not clearly define a response. In this study, 82% of fast progressing participants had a 30% or more reduction in AL growth rate in at least one eye in the first six months. By determining baseline AL growth rates, the response to treatment can be determined accurately.

In the current study, participants continued to receive atropine 0.01% if they demonstrated significant progression after the two years. Some participants have been followed up to 4 years on treatment and continue to tolerate it well and significantly reduced progression rate compared with baseline. A small group have ceased treatment as AL progression has continued to decline to under 0.05 mm per 6 months. This group has been monitored after stopping treatment for a six-month interval. If AL progression increased to greater than 0.10 mm in the intervening six-month period, then atropine 0.01% therapy was resumed. Finally, based on the recent LAMP results, 14 children have recently been changed to atropine 0.05% due to an inadequate response of 0.01% based on AL change. These subjects will be followed longitudinally to determine the treatment effect.

The study has several limitations to be considered. It was conducted in a real-world clinical practice setting limited by randomization to the treatment group, and the examiner was not masked. There was no placebo control group since atropine has been established as an effective intervention. It was not seen as ethical to withhold treatment on children showing fast myopic progression, nor would it be acceptable to their parents. The fast progressors were the treatment group, and there is the possibility that there was some regression to the mean with reduced AL progression not due to the medication. However, these subjects demonstrated a reduction in the progression rate compared to pre-treatment at each time point, while the slow progressors remained steady.

Additionally, the dioptre change had a statistically significant reduction in progression over time which was extended over the treatment period. Another possible confounder is that growth is age-dependant with AL progression, slowing down with age. Although this has been reported, it is essential to point out that the marked reduction was within six months of initiating treatment and the treatment group had a broad age range. There was no control for outdoor exposure or near work for participants; however, all participants were counselled regarding lifestyle choices known to affect myopia progression rates, both prior and during treatment reviews. Further reviews at the defined six-month interval have been difficult to continue due to COVID-19 restrictions. The use of AL measurements could be subject to inter-test variability. However, the reported reproducibility of IOL Master scans in children is high, and the threshold for intervention used in this study of 0.1mm per 6 months is outside the 95% confidence intervals reported in two separate studies conducted in children [36, 37]. Finally, compliance with the atropine was self-reported.

In conclusion, this study representing the experience of a single surgeon paediatric clinical practice demonstrates that measuring a baseline AL growth rate is helpful to identify faster-progressing myopes who should then benefit from atropine 0.01%. The study confirms the beneficial effect of atropine in this group of children of a mixed ethnic Australian population. Furthermore, it highlights that AL is an important biomarker of treatment effect in the clinical setting that can be used in conjunction with other clinical measures. Longer-term follow-up effects and review of treatment benefit in slow progressors still needs to be determined.

## Supporting information

S1 File (XLSX)

S2 File (XLSX)

S3 File (XLSX)

S4 File (XLSX)

S5 File (XLSX)
